# Contemporary Practices for Management of Subclinical Atrial Fibrillation

**DOI:** 10.3390/jcm14155222

**Published:** 2025-07-23

**Authors:** Buthainah Alhwarat, Omar Darwish, Sai Nikhila Ghanta, Aakash Rana, Nitesh Gautam, Subhi J. Al’Aref, Subodh Devabhaktuni

**Affiliations:** 1 Department of Internal Medicine, University of Arkansas for Medical Sciences, Little Rock, AR 72205, USA; balhwarat@uams.edu (B.A.); odariwsh@uams.edu (O.D.); 2 Division of Cardiology, Department of Internal Medicine, University of Arkansas for Medical Sciences, Little Rock, AR 72205, USA; snghanta@uams.edu (S.N.G.); ngautam@uams.edu (N.G.); sjalaref@uams.edu (S.J.A.); 3 Department of Internal Medicine, Central Arkansas Veterans Healthcare System, Little Rock, AR 72205, USA; aakash.rana@va.gov

**Keywords:** subclinical atrial fibrillation, atrial high-rate episodes, stroke prevention, device-detected atrial fibrillation

## Abstract

Subclinical atrial fibrillation (SCAF) episodes are frequently detected in patients with cardiac implantable electronic devices (CIEDs). These asymptomatic arrhythmias are increasingly recognized as potential harbingers of clinical atrial fibrillation and thromboembolic events. However, the management of SCAF—particularly regarding the use of oral anticoagulation (OAC)—remains controversial. This literature review (Medline, Scopus, Goggle scholar, Embase) focuses on using current literature and clinical studies to guide decision-making regarding anticoagulation therapy and other treatment options that can limit complications for patients with SCAF. The decision to initiate anticoagulation in patients with atrial high-rate episodes (AHREs) should be individualized, balancing stroke risk against bleeding potential. Ongoing research and post hoc analyses will further clarify which subgroups may benefit most from therapy, informing future guideline recommendations.

## 1. Introduction

Atrial fibrillation (AF) is arguably one of the most common sustained cardiac arrhythmias, with an estimated overall prevalence of 3–6 million individuals in the United States [1]. Moreover, AF has been associated with an increased risk of downstream adverse cardiovascular events, including a 2.4-fold increased risk of stroke. Over the past decade, the advent of remote monitoring for cardiac rhythm via cardiac implantable electronic devices (CIEDs), event monitors and, more recently, remote wearables has allowed clinicians to hone our understanding of atrial fibrillation. It is now known that the spectrum of atrial fibrillation exists on a continuum. Prior to manifest clinical atrial fibrillation, there is evidence of electrical rhythm changes such as shorter bursts of atrial tachycardia or atrial ectopy, which are predisposing markers for progression to clinical atrial fibrillation [2]. These rhythm changes have been grouped into atrial high-rate episodes (AHREs) and subclinical atrial fibrillation (SCAF).

While atrial high-rate episodes (AHREs) are defined as asymptomatic episodes of atrial tachyarrhythmia that are only detected by CIED, subclinical atrial fibrillation (SCAF) is defined as short episodes of atrial fibrillation without symptoms that are detected by CIED or continuous ECG monitoring without prior diagnosis of AF [3]. The European Heart Rhythm Association (EHRA) defines AHRE as any atrial tachyarrhythmia with an atrial rate of more than 190 bpm detected only by a CIED that lasts for 5 to 6 min [4,5,6]. Generally speaking, SCAF is used interchangeably with AHRE; however, AHRE is a broader term that includes other supraventricular tachyarrhythmias like atrial flutter and atrial tachycardia [2].

While ample data exists to support management of clinical atrial fibrillation, there is a paucity of data for management of patients with SCAF. Through this review, we aim to summarize the current evidence and weight behind the epidemiology and management strategies for AHRE and SCAF.

## 2. Epidemiology and Risk of Thromboembolism

**Figure 1:**
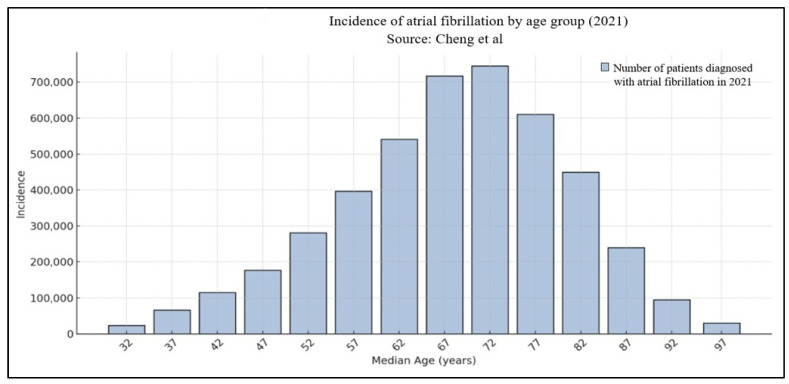
Age-stratified distribution of atrial fibrillation [7].

The risk of clinical AF increases with age and thus the prevalence is constantly rising. For example, in community-based cohort studies conducted in the United States and Europe, the lifetime risk of AF by the age of 80 is estimated to be around 24% for men and 22.5% for women [5]. Figure 1 shows age-stratified incidence of AF in 2021 according to Cheng et al. [7].

Given the risk of stroke in patients with clinical AF, oral anticoagulation (OAC) or left atrial appendage occlusion is recommended for patients who are intolerant to OAC therapy. Studies have shown a measurable albeit lower risk of stroke in patients with SCAF (1.2–2.5-fold) when compared to clinical AF (4-fold) [2,8].

**Figure 2:**
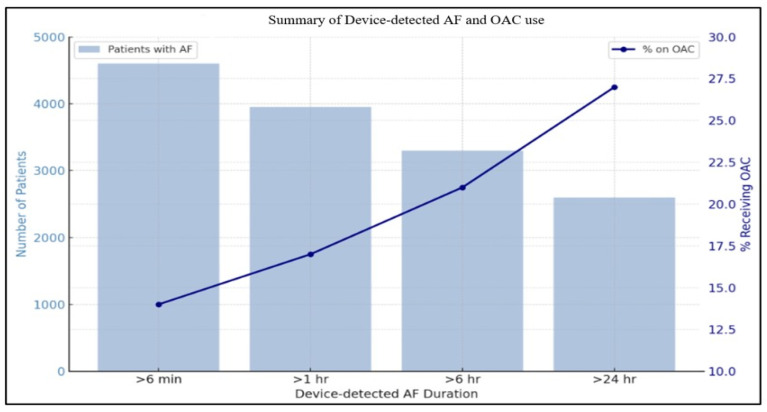
Summary of device-detected AF and OAC use.

Despite knowledge of the measurable risk of stroke in this population, there is a significant variability in clinical practice regarding OAC initiation in patients with device-detected atrial fibrillation. For instance, in a retrospective study conducted by Perino et al., there was a significant difference in the rates of OAC prescription in SCAF. While the prescription rate was 16% in individuals with SCAF duration >1 h, the rates only reached 27% amongst patients with SCAF duration >24 h, which has been demonstrated in the ASSERT (The Asymptomatic Atrial Fibrillation and Stroke Evaluation in Pacemaker Patients and the Atrial Fibrillation Reduction Atrial Pacing Trial) trial to be associated with a higher risk of stroke (Figure 2) [8,9].

Moreover, trials such as TRENDS and ASSERT have established arrhythmic burden—defined by the cumulative duration and frequency of subclinical atrial fibrillation (SCAF) episodes—as a stronger predictor of thromboembolic events than isolated, single-episode duration. In the TRENDS study, patients with a daily SCAF burden exceeding 5.5 h had an annual stroke risk of approximately 2.4%, more than double that of those with lesser burden [4]. On the other hand, ASSERT, which followed 2455 pacemaker/ICD patients over 2.5 years, categorized the longest SCAF episodes: 18.8% experienced episodes lasting over 6 min to 6 h, 6.9% had durations of 6–24 h, and 10.7% had episodes longer than 24 h [10]. Most notably, episodes > 24 h were linked to a significant 3.24-fold increase in risk of ischemic stroke or systemic embolism (adjusted HR 3.24; 95% CI 1.51–6.95; *p* = 0.003). Meanwhile, shorter episodes (<24 h) were not associated with statistically significant elevated risk. These findings underscore that overall SCAF burden, not just the longest individual episode, is a powerful independent prognostic marker for thromboembolic events.

In addition, a growing body of evidence supports the concept of atrial cardiomyopathy (ACM) as a central contributor to the development and progression of AF, including its subclinical forms. ACM is defined as any complex of structural, architectural, contractile, or electrophysiological changes affecting the atria, with or without clinical manifestations such as arrhythmia. These alterations, which may result from systemic conditions like hypertension, diabetes, obesity, or sleep apnea, can create a pro-arrhythmic atrial substrate characterized by fibrosis, inflammation, conduction abnormalities, and atrial mechanical dysfunction. Such remodeling increases susceptibility to AF by facilitating both the initiation and maintenance of abnormal atrial electrical activity.

Recent studies have demonstrated that ACM can precede the first detectable episode of AF, implying that atrial remodeling may silently progress for years before overt arrhythmia is documented. This is particularly relevant for SCAF and AHREs, which are frequently detected via cardiac implantable devices or advanced wearable monitors in patients with no overt symptoms. In such cases, the atrial structural substrate may already be significantly altered despite the absence of traditional clinical markers of AF [11].

Moreover, the identification of atrial cardiomyopathy through advanced imaging modalities—such as cardiac magnetic resonance imaging (MRI) with late gadolinium enhancement, speckle-tracking echocardiography for left atrial strain, or voltage mapping during electrophysiology studies—offers the potential to stratify patients at risk for future AF development and associated thromboembolic complications. Genetic studies have also begun to elucidate hereditary patterns of atrial remodeling and AF predisposition, indicating that ACM may, in some cases, have a genetic or familial basis.

## 3. Implantable Loop Recorders and Advanced Monitoring in SCAF

The use of implantable loop recorders (ILRs) and other long-term cardiac monitoring devices has revolutionized detection and management of SCAF, helping to detect asymptomatic AHREs that often elude intermittent monitoring. A retrospective analysis of 64 cryptogenic stroke patients using ILRs demonstrated a high rate of arrhythmia detection—35% overall; specifically, atrial fibrillation, which would be SCAF in this instance, was identified in 25% of cases, over half of which were clinically silent [12].

Moreover, Cersosimo et al. (2025) investigated the predictive value of atrial strain using speckle-tracking echocardiography in stroke patients who received implantable cardiac monitors (ICMs, a category that includes ILRs) [13]. Over a median follow-up of 15.3 months (IQR 7.4–23.5), nearly half (96/204, 47%) of patients experienced SCAF episodes > 6 min. These findings highlight the benefit of ILRs in detection of asymptomatic SCAF which would otherwise go unrecognized.

## 4. Anticoagulation in AHRE/AHRE/SCAF

Current literature shows that there is a significant association between SCAF and strokes as well as systemic thromboembolism events. As shown in the ASSERT trial, CIED-documented AHREs, i.e., SCAF episodes, were associated with an increased risk for ischemic strokes and thromboembolism (TE) events by a factor of 2.5, signifying a large burden of adverse clinical outcomes for these patients [14]. Whether this applies to all SCAF episodes and if the relationship is duration-determined are more nuanced questions which have been attempted to be answered in recent trials. Moreover, a knowledge gap currently exists in what we know about the nature of the utility and benefit of OAC therapy for stroke/TE prevention in these patients [9].

One trial that studied the effect of OAC therapy in patients with SCAF was the ARTESIA (Apixaban for the Reduction of Thrombo-Embolism in Patients with Device-Detected Subclinical Atrial Fibrillation) trial [15]. This double-blinded study added significant value to the existing literature by comparing the effects of treatment with direct oral anticoagulant (DOAC), namely apixaban, versus treatment with aspirin (ASA) in patients with SCAF in terms of risk reduction of ischemic strokes and risk of bleeding. The included patient sample consisted of 4014 patients in whom SCAF was detected through a CIED lasting at least 6 min and up to 24 h. Criteria for selection also included a CHA2DS2-VASc score of 3 or higher and a past medical history free of clinical AF. The CHA2DS2-VASc score (congestive heart failure, hypertension, age, diabetes mellitus, prior stroke or TIA or thromboembolism, vascular disease, age, and sex category) has been used for many years to assess the risk of stroke in patients with clinical atrial fibrillation. Findings of this pivotal trial were significant: stroke risk was lower by 37% in the treatment group with apixaban (0.78% per year, *n* = 55) versus ASA alone (1.24% per year, *n* = 86). Moreover, results also showed a reduced risk of disabling or fatal stroke by 49% in the apixaban group. Of note, in the 82 strokes that were documented during the follow-up period in both patient groups, 37 (45%) were fatal or resulted in significant permanent disability. Noted also was an 80% increased risk of major bleeding in the apixaban arm (1.71% per patient year) when compared to ASA alone (0.94% per patient year) (HR 1.80; 95% CI [1.26–2.57], *p* = 0.001). While the risk of bleeding was higher, apixaban did not result in higher rates of devastating bleeding complications (e.g., intracranial hemorrhage, fatal bleeding, etc.) than ASA [15].

A subgroup analysis of the ARTESIA trial, published later, studied the benefit of OAC or secondary stroke prophylaxis in patients with SCAF. This analysis by Shoamanesh et al. particularly compared the benefit of stroke prevention by OAC therapy or other means in SCAF/AHRE patients with a documented history of a stroke or a transient ischemic attack (TIA). This study documented a 7% stroke/systemic embolism absolute risk reduction associated with the use of the DOAC apixaban versus 1% (ASA group) in SCAF patients with a history of either a stroke or a TIA during a follow-up period of 3.5 years. On the other hand, it delineated an increased bleeding risk of 3% in the DOAC group versus 1% in the ASA group [16].

Another analysis of the ARTESIA trial, conducted by Lopes et al. in 2024 [17], examined the baseline CHA2DS2-VASc score for this randomized controlled trial’s (RCT) participants and addressed the corresponding stroke/systemic embolism and major bleeding risk according to their treatment group. They found that in every patient with a CHA2DS2-VASc score of 4 or higher, apixaban significantly helped lower stroke/embolism risk (0.98%/year, compared to 2.25/year in the aspirin group). They also found that the bleeding risk was higher in the apixaban group, documenting a major bleeding rate of 0.68 in this patient group [17].

Another important trial discussing OAC therapy in AHRE was the NOAH-AFNET 6 trial (Non-Vitamin K Antagonist Oral Anticoagulants in Patients with Atrial High Rate Episodes) (Table 1) [18]. This double-blinded trial assigned 2536 patients randomly to either receive edoxaban or a placebo. Inclusion criteria involved evidence of an implantable cardiac device-documented AHRE/SCAF episode (s) lasting at least 6 min with a heart rate of at least 170 beats per minute. Patients were also chosen based on having at least one additional clinical risk factor for stroke or thromboembolism such as congestive heart failure (CHF), hypertension, age > 75 years old, or history of previous strokes [15]. Of note, patients were excluded from enrollment if they had evidence of prior clinical AF.

The NOAH-AFNET 6 trial was terminated at 21 months of follow-up. Prior to early termination due to concerns related to patient safety, the NOAH-AFNET 6 trial assigned 1270 patients to receive the DOAC edoxaban and 1200 to receive either placebo or 100 milligrams (mg) of ASA (based on prior accepted patient indications). The absolute incidence of strokes was low at 0.9% per patient year (*n* = 22) in the edoxaban group, compared to 1.1% per patient year (*n* = 27) in the control group who received ASA or placebo. Other significant findings of this study included a higher risk of major bleeding (occurring in 53 patients in the edoxaban versus 25 in the placebo group) as well as death (from any cause), which happened in 111 patients in the edoxaban group compared to 94 in the placebo group [15].

**Figure 3:**
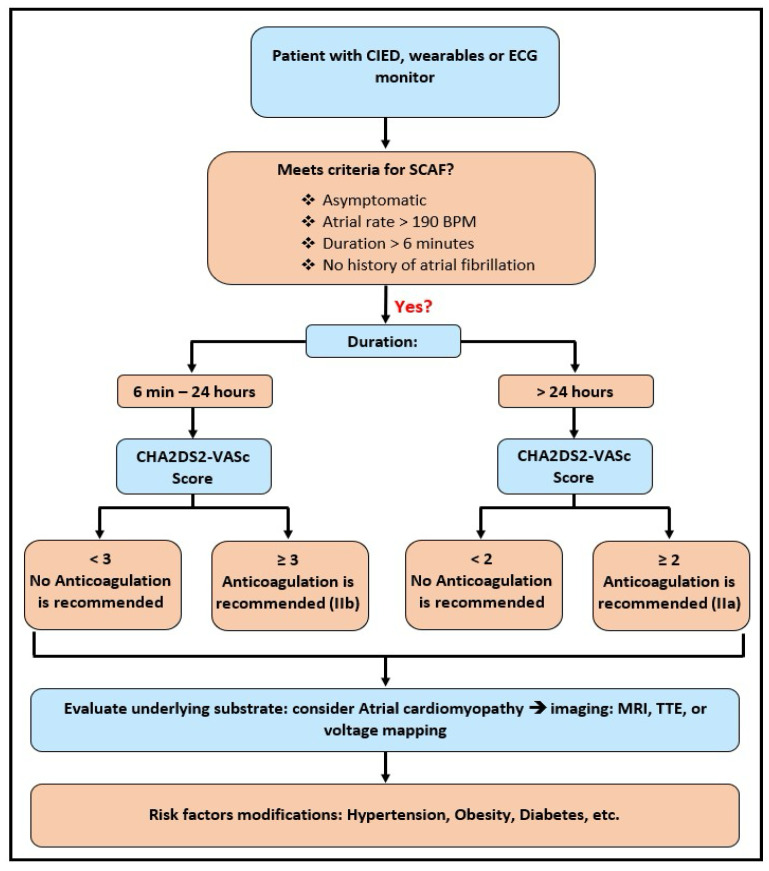
Clinical management algorithm for subclinical atrial fibrillation (SCAF), incorporating episode duration, CHA_2_DS_2_-VASc score-guided anticoagulation initiation, and subsequent evaluation and risk factor modification.

The ARTESIA and NOAH-AFNET 6 trials are both landmark studies in the realm of SCAF and its management. They both documented a lower absolute incidence of strokes and TE events, which can result in catastrophic clinical outcomes in patients. The current American College of Cardiology (ACC) guidelines, published in 2023, not incorporating the evidence from the aforementioned trials, recommend the initiation of OAC therapy in patients with SCAF with episodes lasting equal to or longer than 24 h in the background of a CHA2DS2-VASc score of 2 or higher as a class 2A indication. Moreover, the guidelines also point out that it may be worthwhile to initiate OAC in those with SCAF episodes lasting anywhere from 6 min up to 24 h in the setting of a CHA2DS2-VASc score of 3 or higher as a class 2B indication (Figure 3) [18].

**Figure 4:**
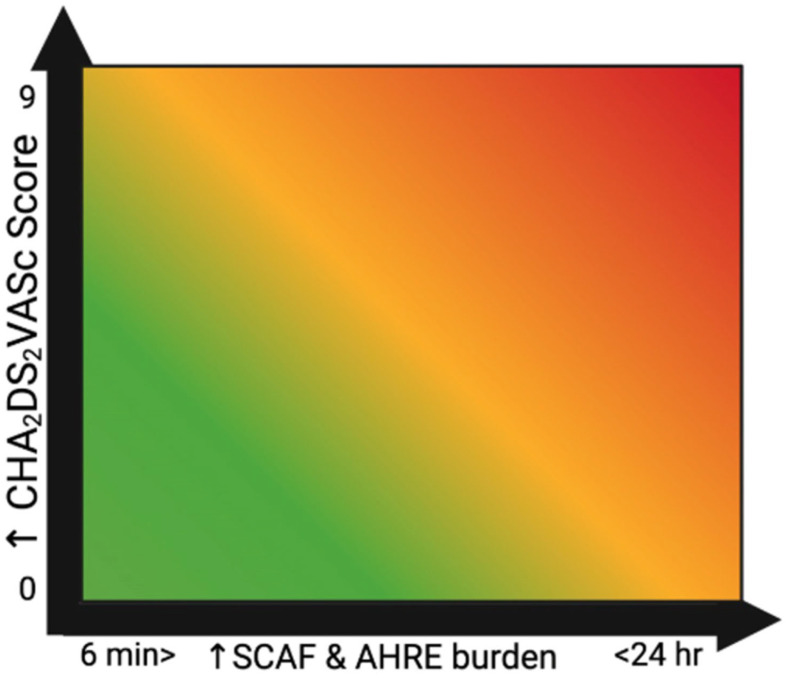
Relationship between SCAF and CHA_2_DS_2_VASc score depicted as a heat map.

Therefore, and in light of the corresponding RCTs, it appears that a large subset of SCAF patients would have a morbidity and mortality benefit from OAC therapy, especially when their episodes are long-lived and in the setting of other stroke/TE risk factors coexisting with their SCAF (Figure 4). However, the results of these trials show that OAC therapy with DOAC agents has produced inconsistent results. Therefore, further research is needed to determine which specific DOAC agents are most appropriate for TE prophylaxis in patients with SCAF.

Despite heavy research on the subject, many areas remain ambiguous when it comes to anticoagulation therapy in SCAF. Does this risk reduction remain clinically significant when adjusting to other stroke risk factors and do the benefits of therapy with DOACs outweigh their risks of potentially life-threatening side effects, including but not limited to major bleeding? Additional similar RCTs are indeed required to address these current gaps in knowledge to help better assess these patients’ stroke risk and guide treatment accordingly. Left atrial appendage occlusion devices might be an option in subsets of patients with SCAF, like those with a history of prior stroke in whom risk of bleeding with long-term use of OACs can be mitigated. RCTs studying this approach are needed to have a definitive answer.

## 5. Risk of Progression to Clinical Atrial Fibrillation

When addressing the complexity of identifying and managing AHRE/SCAF, its potential to convert into overt, clinical AF is no exception to the rule. The MOST (MOde selection trial) RCT, published in 2003, defined AHRE as episodes of a heart rate faster than 220 bpm lasting at least 5 min and had a total of 2010 patient participants in the main trial who were assessed for SCAF incidence and the development of symptoms that can be attributed to AF. Through this RCT, the authors documented an increased risk for progression to overt clinical AF based on symptom development in patients who had evidence of device-documented AHREs [19]. However, this study was limited by the fact that around 60% of participating patients had a history of supraventricular tachycardia (SVT) prior to enrollment, which was not further specified. Therefore, one may argue there was not enough evidence to support that the ECG evidence of AF, which was documented in a portion of the participants in this study, was not just merely a recurrence of previous AF.

On the other hand, Wong et al. analyzed the data extracted from the ASSERT (Asymptomatic Atrial Fibrillation and Stroke Evaluation in Pacemaker Patients and the Atrial Fibrillation Reduction Atrial Pacing Trial) trial, which only included patients with no past medical history of AF [20]. They found that out of 415 patients with evidence of SCAF/AHRE, 65 patients (15.7%) went on to develop either clinical AF (25 patients) or SCAF lasting > 24 h (60 patients) or both (20 patients) [20]. This study also documented a pattern of association between SCAF progression (into prolonged episodes or clinical AF) with several risk factors as observed by the data from the ASSERT trial. These risk factors included advanced age, increasing body mass index (BMI), and length of SCAF episodes, with every 1 h increment resulting in approximately 13% increased risk of SCAF progression. The results of this study also demonstrated a consistent increase in the median duration of SCAF episodes in the patients who experienced SCAF progression. Specifically, the longest-lived SCAF episodes in these patients increased from 6.7 h in the first year of follow-up to an average of 153.2 h during the fourth year of follow-up. In contrast, the median duration of the longest-lived SCAF episode remained unchanged for patients who did not experience SCAF progression. This finding is clinically relevant because it comes with the conclusion that patients who have SCAF progression to either > 24 h long episodes or overt AF likely have an underlying substrate predisposing them to SCAF progression and thus worse clinical outcomes.

Van Gelder et al. documented that SCAF duration of over 24 h (found in 11% of study participants at 3 years of follow-up) is associated with adverse clinical outcomes, specifically stroke and TE risk. They found that in patients who had SCAF progression, their risk of stroke and systemic embolism was 3.1% per patient year as compared to 0.5% in patients who did not undergo SCAF progression, signifying a substantial difference in risk for adverse events [10].

It has been established by many studies in existing literature that there is an incremental relationship between the length of AF (clinical variant) episodes and an increased stroke risk [21]. In light of Van Gelder’s findings, it also appears that the same applies to SCAF episodes lasting > 24 h, which fall under the term “SCAF progression.” This raises the following question: what is the role of rate and rhythm control, the conventional treatment for clinical AF, in these patients with SCAF, especially those who go on to develop prolonged SCAF episodes?

While there is a reasonable amount of evidence of the benefit of OAC therapy in a subset of SCAF patients, the same unfortunately does not apply to rate and rhythm control therapies. Current guidelines do not address a large component of this burning issue. Some may argue there may be benefit for baseline rate control for patients with SCAF; however, taking its episodic nature into account, with many patients having episodes lasting only a few minutes, might defeat the purpose of rate control in a lot of ways. The most recent ACC/Heart Rhythm Society (HRS) guidelines on AF only discuss the need for acute rate control in episodes of rapid ventricular response with an HR over 100 bpm in conditions that fall under the “regular” AF definition [18].

In regard to rhythm control, existing studies in current literature, which are largely observational, document a benefit of catheter ablation as rhythm control therapy when it comes to preventing the progression of paroxysmal AF, which some may argue is similar to SCAF, at least in terms of intermittency [18]. One specific study by Kim et al. featured patients with asymptomatic AF in a prospective observational manner. They assessed patients’ response and clinical outcomes following rhythm control therapy with either catheter ablation alone or combined with chemical cardioversion or that performed with cardioversion (chemical or electrical) alone [22]. They found that patients with asymptomatic paroxysmal AF benefited from rhythm control therapy and experienced overall better clinical and cardiovascular outcomes when compared to patients with symptomatic AF [22]. Specifically, they documented better ischemic stroke, hospitalization secondary to heart failure, and cardiac death rates in this patient group. Factors favoring desirable outcomes in the asymptomatic patient sample included a left atrial diameter of 50 mm or less and a CHA2DS2-VASc score of 3 or higher. However, despite how “similar” paroxysmal AF and SCAF might be, this does not inherently mean that the results of these studies can be generalized to apply to patients with SCAF. As rhythm control therapy, through the use of an antiarrhythmic agent, electrical cardioversion, or catheter ablation, comes with its own risks, there are multiple considerations to take into account prior to considering it as an option for patients with mere SCAF. Going forward, there ought to be formal clinical studies conducted strictly on patients with evidence of SCAF to evaluate the role of rate and rhythm control therapy and its long-term benefits and risks to help improve cardiovascular outcomes for these patients. While existing observational studies suggest that early rhythm control—including catheter ablation—may improve outcomes in asymptomatic clinical AF, particularly paroxysmal AF, it remains unclear whether the same benefits extend to patients with SCAF or device-detected AHRE. The management of SCAF presents unique challenges, as these episodes are often brief, asymptomatic, and detected only through continuous monitoring devices rather than clinical presentation. Unlike asymptomatic AF identified on ECG, which may still prompt rhythm control in select patients, current guidelines do not routinely recommend ablation for isolated AHREs or SCAF in the absence of additional risk factors or symptom burden. Future randomized studies are needed to determine if early intervention in this population can reduce progression to sustained AF or adverse cardiovascular outcomes.

Wearable sensors such as ECG patches, seismocardiogram-based monitors, and photoplethysmography (PPG) wristbands are increasingly being integrated with AI/ML models to identify arrhythmias, assess cardiac function, and predict adverse cardiac events. These technologies offer continuous, non-invasive monitoring and have demonstrated promising accuracy in detecting early signs of decompensation and arrhythmogenic episodes. For example, AI-driven analysis of ECG and heart rate variability data has enabled earlier detection of atrial fibrillation, while predictive models using supervised learning (e.g., Random Forest, SVM) have shown strong performance in risk stratification of sudden cardiac death and high-rate atrial episodes [23].

Moreover, time-series forecasting models using recurrent neural networks (RNNs), including long short-term memory (LSTM) and gated recurrent unit (GRU) architectures, are being developed to anticipate physiological changes that precede SCAF episodes [23,24]. These models can leverage longitudinal data from wearables to identify high-risk individuals even before clinical symptoms emerge. Incorporating these technologies into the SCAF management algorithm provides a more proactive and personalized approach, potentially improving early detection and guiding timely interventions.

## 6. Heart Failure and SCAF/AHRE

Heart failure with preserved ejection fraction (HFpEF) and AF can, very commonly, coexist in a lot of patients [25]. This combination is known to cause an exponentially higher burden of morbidity and mortality in patients. The RELY (Randomized Evaluation of Long-Term Anticoagulation Therapy) trial revealed that patients with AF are much more likely to die from congestive heart failure (CHF)-related complications versus an ischemic stroke, accounting for 15.1% of deaths versus 7.0%, respectively [26]. The nature of this cause-and-effect relationship has been well described by existing literature. AF is a common cause of CHF through tachycardia-induced cardiomyopathy and the resulting cardiac remodeling, and vice versa. CHF can precipitate AF through elevated intracardiac pressures and dysfunction of the autonomic and neuroendocrine pathways, among multiple other causative mechanisms [26]. That being said, there exists a gap in how much we know about the relationship between CHF and SCAF. Is it a similar relationship? Can the same management approach be applied to these patients?

Yang et al. performed a pioneering study regarding the combination of CHF and SCAF [27]. He compared the incidence of SCAF in patients with HFpEF versus that in a patient pool without HFpEF and found that the patient group with HFpEF had over double the incidence of SCAF (8.9%) compared to those without (4.1%). They also found that following risk factor adjustment, the risk of SCAF in the setting of HFpEF was even higher with an odds ratio (OR) of 3.01 (95% CI: 1.13–7.99; *p* = 0.03) [27]. Moreover, a study published by Nishinarita et al. monitored 104 patients without a past medical history of clinical AF with implantable cardiac devices for the development of SCAF/AHRE (monitored for one year) as well as symptoms of CHF over a follow-up period of seven years [28]. Despite not addressing the number of patients who experienced new-onset CHF, this study delineated an important association between the development of AHRE and CHF (defined as worsening of an existing CHF), as out of the 34 patients who had new-onset AHREs, 23.5% had evidence of worsening CHF during follow-up [28].

On the other hand, Kahwash et al. analyzed data obtained from ICM devices of CHF patients between the years 2007 and 2021. They found that patients who developed device-documented AF, i.e., SCAF, had a higher incidence (27%) of CHF events when compared to patients without documented AF episodes (13.7% incidence). This patient cohort was also found to have a higher all-cause death rate (18.9%) [29].

Moreover, an additional study by Patel et al. evaluated 367 patients with HFpEF (diagnosed through evidence of diastolic dysfunction on echocardiogram) and CHF with mid-range ejection fraction, also known as HFmrEF, defined by LVEF between 40% and 50% via echocardiography, for evidence of SCAF [30]. Results of this study were significant for an incidence of nearly 40% of device-detected SCAF in patients with HFpEF and HFmrEF over a timeline of one year of follow-up.

In light of these findings, it is evident that the subclinical variant of AF, which is exclusively detected in an incidental fashion, also shares a bi-directional relationship with CHF. These studies demonstrate a clear association between the two conditions, with patients suffering from either SCAF or CHF having a higher likelihood of developing, or experiencing a greater morbidity burden from, the other condition. This also translates to worse cardiovascular outcomes, including higher major adverse cardiovascular event rates, and overall clinical outcomes for a significant subset of SCAF patients.

## 7. Controversies and Challenges

As mentioned above, AHREs have been associated with increased risk for both stroke and development of clinical AF. The main question remains whether to initiate anticoagulation or not. When looking at the available evidence, there is no justification for the use of routine AC in patients with AHREs; however, other modifiable risk factors for stroke and clinical AF should be addressed and managed accordingly [31]. It is evident that stroke risk depends not only on the burden of AHREs but also on other risk factors for stroke, namely the CHA2DS2-VASc score. It is also suggested to start AC if the burden of AHREs is more than 24 h, but no recommendations are mentioned regarding the appropriate management of AHREs with a burden of less than 24 h. Moreover, while existing RCTs have documented improved outcomes for SCAF patients who received OAC therapy with DOACs, further research is needed to contrast clinical outcomes for these patients with different DOAC agents for the best estimation of their safety profile in this patient group. Hopefully, the ongoing RCTs (NCT 01938248, NCT 02618577) may provide evidence on the correct management of this cohort [32].

## Figures and Tables

**Table 1:** Outcomes of OAC in SCAF compared using data from NOAH-AFNET 6 and to ARTESIA study.

Study Comparison:		
	NOAH-AFNET 6 vs.	ARTESIA
Year	2023	2023
Inclusion criteria	At least one episode of device-detected SCAF lasting at least 6 minutes with a heart rate of at least 170 bpm + At least one other stroke risk factor like age, CHF, etc.	At least one episode of device-detected SCAF lasting at least 6 minutes up to 24 hours + Age of 55 or older + No history of clinical AF.
Study groups	Edoxaban Vs Placebo	Apixaban Vs Aspirin (ASA)
Sample Size	2608 (1:1)	4012 (1:1)
Mean CHA2DS2-VASc score	4	3.9
Median follow up (months)	21	42
Mean age (years)	78	76.8
Median duration of AHREs (hours)	2.8	1.47
Results	Higher bleeding in Edoxaban group with no significant difference in stroke & thromboembolism	Apixaban reduced the risk of stroke & thromboembolism significantly compared to ASA alone but increased the risk of major bleeding
Limitations	Study terminated early due to safety concerns. Confounding bias due to presence of multiple stroke risk factors in patients enrolled in the trial.	Results can only be extrapolated to patients with existing risk factors for stroke/systemic embolism (confounding bias).

## Data Availability

No new data were created as the article serves as a literature review for the current evidence.

## Abbreviations

The following abbreviations are used in this manuscript:

AC	Anticoagulation
CIED	Cardiac Implantable Electronic Device
CHF	Congestive Heart Failure
SCAF	Subclinical Atrial Fibrillation
AHRE	Atrial High-Rate Episode
